# Improving access to medicines and beyond: the national volume-based procurement policy in China

**DOI:** 10.1136/bmjgh-2022-011535

**Published:** 2023-07-18

**Authors:** Zheng Zhu, Quan Wang, Qiang Sun, Joel Lexchin, Li Yang

**Affiliations:** 1 Department of Health Policy and Management, Peking University School of Public Health, Beijing, China; 2 Brown School, Washington University in St. Louis, St. Louis, Missouri, USA; 3 Shandong University School of Public Health, Jinan, Shandong, China; 4 Department of Family & Community Medicine, University of Toronto, Toronto, Ontario, Canada; 5 School of Health Policy and Management, York University, Toronto, Ontario, Canada

**Keywords:** health policy

## Abstract

Since 2019, the Chinese central government has taken significant steps to centralize national purchasing power and has implemented a pooled procurement system. In this paper, we provide an in-depth analysis of China's National Volume-Based Procurement (NVBP) policy, which represents a unique approach to pooled procurement within the pharmaceutical sector. The primary objectives of the NVBP are to reduce drug prices, enhance access to affordable medications, and improve the overall functioning of the pharmaceutical industry in China. Our analysis delves into the key features of the NVBP, including its centralized procurement system, volume-based procurement approach, and the guaranteed procurement volumes allocated to winning bidders. We also address the challenges and implications associated with the NVBP, such as its impact on the pharmaceutical industry, the sustainability of price reductions, and the importance of striking a balance between price reduction and industry sustainability. Through a comparative analysis, we shed light on the distinct characteristics of China's approach to pooled procurement and its potential ramifications for healthcare policies and practices. By examining the NVBP within the broader context of China's evolving healthcare landscape, we aim to contribute to a deeper understanding of the implications and effectiveness of this unique policy initiative.

**SUMMARY BOX**

- National volume-based procurement increases accessibility and affordability of common drugs in China.
- Clear, guaranteed expectations of large markets spur the dynamism of pharmaceutical companies and promote sustainable market competition.
- A fair, free and transparent institutional structure is essential for the trust and relationships among government, pharmaceutical manufacturers and the public.

Price directly impacts accessibility to medicines, particularly in low and middle-income countries (LMICs), where a substantial proportion of drug spending is borne by out-of-pocket payments and drug availability through the public sector is relatively weak.^1^ The strategic purchasing of medications has been identified as a potentially promising driver to facilitate an effective healthcare system that consistently delivers affordable drugs, improves health outcomes and responds to changing healthcare demands of the local population.^2^ From 2018 to 2022, China has implemented seven rounds of national volume-based procurement (NVBP) that have reduced the prices of 294 formulations of multiple drugs by an average of 53%. China’s experience has shown that pooling purchasing power can be a powerful tool for LMICs to gain bargaining advantages, improve access to medicines, create a competitive market and ultimately improve health outcomes.

## The evolution of the NVBP

**Figure 1:**
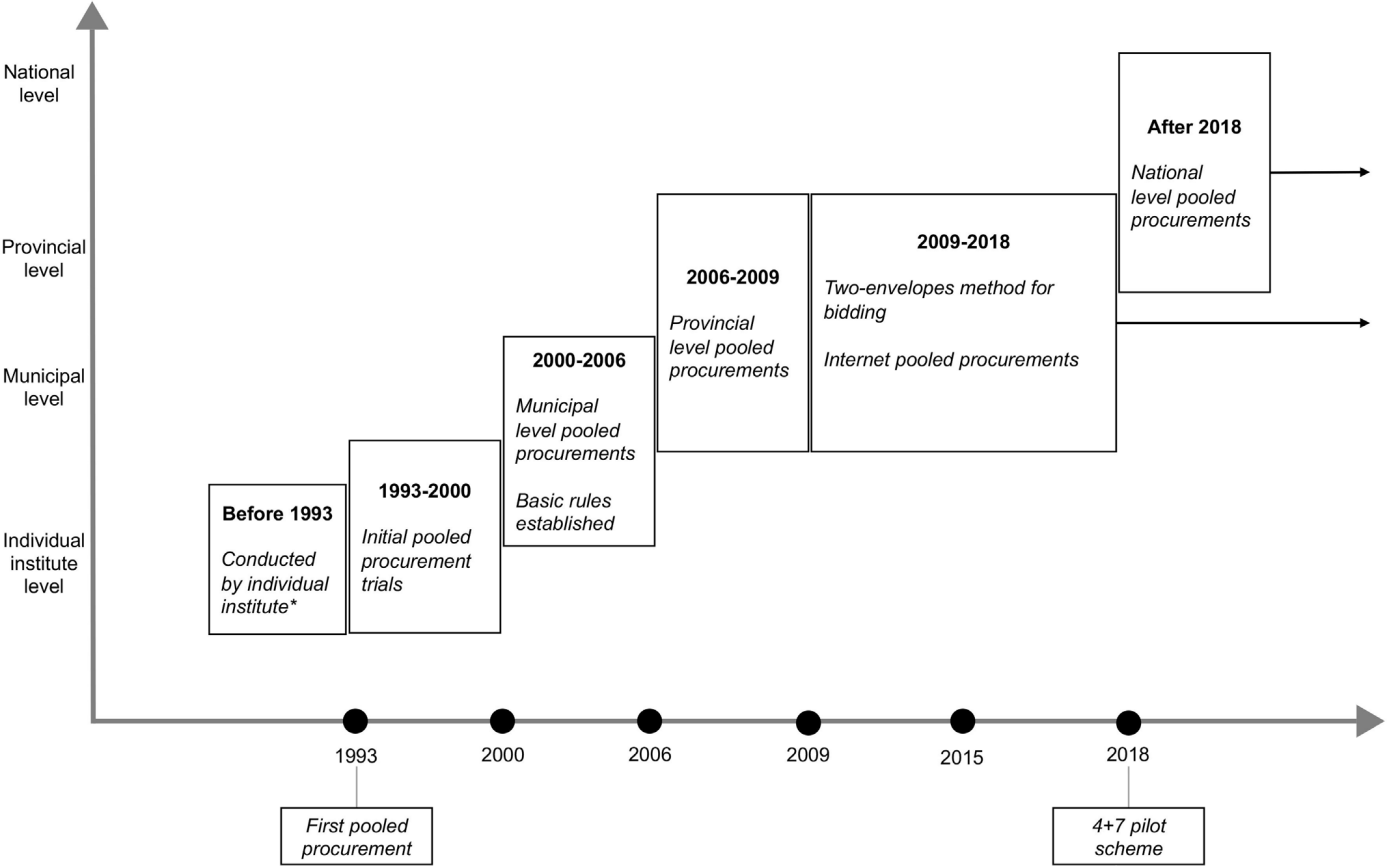
Timeline of China’s drug procurement policies. Source: Self-made by the authors of this study. *Any facility that provides healthcare services such as hospitals, primary care institutes and public health centres.

Since 1993, Chinese local governments have been able to centralise medicine purchasing power and facilitate pooled procurement within their regions.^3^ One clear trend in policy implementation is that the level of centralisation has continually increased (figure 1). Initially, in 1993, the first pooled procurement initiative involved only several public hospitals. In the following decades, the policy has evolved from the municipal to the provincial level and eventually reached the national level. The motivation behind this policy evolution was twofold: to reduce drug prices and to eliminate bribery from the procurement process. The former is a common reason for pooled procurement in many other countries, while the latter is specific to the non-transparent and potentially corrupt way that China’s public hospital drug market was accessed and the relationship between the Chinese government and industry.

The next step in the evolution of the procurement process was the introduction of the *Tendering and Bidding Law* of 1999. Prior to this law, there were mainly three methods for drug procurement at the local government level: public tendering, invited tendering and price negotiation. The enactment of the law was a significant milestone and since then public tendering has become the most popular and is widely used by local governments. The local experiments and practices established the basic rules and patterns for public drug procurement, many of which are still in place today, such as the two-envelope method, internet pooled procurement and categorised procurement (box 1).

Box 1 **China’s experience with drug procurement**

**Two-envelope method**

The two-envelope method, a uniquely Chinese method, has been widely used in tenders, especially those conducted by government. The information from all bidders will be divided and enclosed into two anonymous envelopes: the first one contains all the technical details and the second one is about price. The bidding usually has two rounds. In the first round, an expert group assembled by the local government will open the first envelope and determine qualified bidders, based on independent scoring by the experts. In the second round, the expert group will open the second envelope (only the ones from qualified bidders) and determine the winner, which usually is the one with lowest price.

**Internet-pooled procurement**

The local government typically operates an online system for pooled drug procurement that offers comprehensive information on the tender, such as the commodity being sought, the timeline, bidder qualifications, and the bidding process and the eventual winner is announced through the online system. In certain regions, the system also serves as an online trading platform.

**Procurement of different categories of goods and products**

For different categories of medications or medical devices, the government usually tends to use a different procurement method, like the volume-based procurement, which is typically used for common drugs with large markets covered by public plans and the National Reimbursement Drug Price Negotiation, which is used for new drugs.

However, the drug market was still not fully free, transparent and competitive and became much more complicated as its size rapidly expanded. One major flaw of provincial-level pooled procurement was that local governments were merely agents through which public purchasing power operated, but the actual power was held by medical institutions. After public tendering, drug prices were determined without any clarity about the quantity being ordered, and suppliers had to negotiate with medical institutions to sign specific contracts (a process known as secondary negotiation) about drug prices and quantity.^4^ In practice, the prices of drugs, after the completion of secondary negotiations, were slightly lower than those determined by public tendering. The price difference indicates that pooled procurement did not fully integrate centralised purchasing power with public tendering, as the actual purchasing price was determined through secondary negotiation. Local governments had difficulty regulating every individual medical institution and bribery impeded market competition. Additionally, the fulfilment of signed contracts was also problematic, with many suppliers experiencing delayed payment or changes in the quantity being ordered.

In 2018, with strong political commitment and key leadership, China’s central government announced an NVBP policy. The first round of the NVBP involved four provincial-level cities and seven subprovincial-level cities (the 4+7 pilot scheme). One year later, all provincial entities in mainland China engaged in the NVBP. It is worth noting that even though the NVBP has become the mainstream method for public procurement, the previous pooled procurement method at the provincial level still exists and functions in China.

## How the NVBP functions

**Figure 2:**
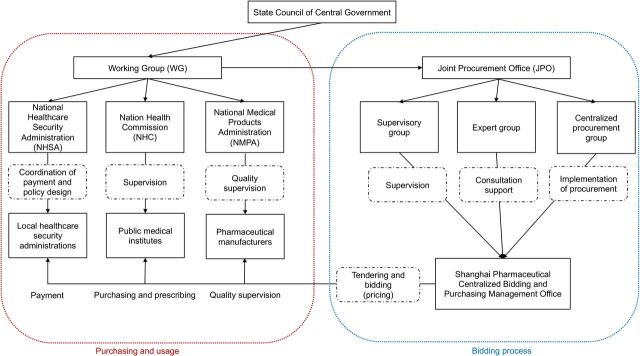
Structure of China’s National Volume-Based Procurement policy. Source: Self-made by the authors of this study.

The structure of the NVBP links bidding, purchasing and use (figure 2). The working group (WG) is responsible for the regulation, evaluation and organisation of the NVBP programme. It also helps to coordinate the relationships among different government departments and different regions. Under the management of the WG, the joint procurement office (JPO) consists of representatives from participating jurisdictions who are responsible for conducting pooled procurement and overseeing the implementation of NVBP results on behalf of the involved public medical institutes. Within the JPO, three groups have been established: the supervisory group, which oversees the bidding process and handles any potential complaints; the expert group, which provides consultation support on policy, clinical guidance and procurement and the centralised procurement group, which is responsible for the administrative implementation of procurement. The Shanghai Pharmaceutical Centralized Bidding and Purchasing Management Office is responsible for the day-to-day operations of the JPO. Only manufacturers of drugs passing the generics consistency evaluation (GCE), that is, bioequivalent drugs, are qualified to enter the bidding. During the bidding process, the JPO considers price as the most important criterion, leaving the manufacturer(s) with the lowest price(s) as the winner(s). By using their administrative authority, the central and local governments regulate all purchasing and usage (but not the bidding process, where the JPO collects all of the required quantities from individual hospitals and other facilities) on both the supply and demand side. The individual medical facilities, primarily hospitals, function as the main payers when procurement contracts are signed.

The design of the NVBP helps address the problems of corruption and inefficiency that were created by a fragmented pharmaceutical system in China. By centralising purchasing power, the NVBP allows for more standardised and transparent procedures and reduces opportunities for corruption. Furthermore, the standardised process for bidding and procurement across the country reduces the need for individual provinces or hospitals to negotiate with multiple suppliers, which can be time-consuming and inefficient. By centralising the procurement process, the government can better monitor and track drug supplies, identify potential supply chain disruptions and take proactive measures to ensure the availability of essential drugs.

### Consistent evaluation of generics

Before 2004, there were two types of marketing licenses for drugs: those approved by the previous national or local Food and Drug Administration. Drugs with the former could be sold across the entire Chinese mainland, whereas those with the latter could only be sold in the local region where they were approved. Due to poor regulation, many locally approved drugs were deemed to be of poor quality. From 2001 to 2004, the central government managed to merge local and national marketing licenses into a new national marketing license. Although some locally approved drugs were taken out of the market, some drugs of unreliable quality still received national marketing licenses and are being sold.

Compared with similar policies in other countries, the GCE is a distinguishing feature of the NVBP. The GCE mainly tests two aspects of generic drugs in comparison to their originators: in vitro pharmaceutical equivalence and in vivo bioequivalence, which ensures that domestically produced generic drugs are identical to their corresponding brand name versions. The GCE excludes drugs of unreliable quality from the NVBP, remedying the problem of merging the two licensing systems and ensuring quality within the public hospital system. Only the drugs that have passed the GCE can participate in the NVBP bidding. Therefore, factors besides price and stability of supply do not need to be considered, which simplifies the tendering process and makes standardised and transparent procedures possible. Although the GCE is not formally a step in the NVBP, it de facto functions as ‘step 0’.

### Clear and guaranteed market expectations

Another key aspect of the NVBP policy is its guarantee of precise procurement volumes for winning bidders, particularly the primary winner. In the past, the provincial pooled procurement system faced challenges in effectively aligning the incentive for lower prices with procurement volumes. This lack of alignment was primarily due to either not being able to clearly estimate the size of the market, which is only finally determined through secondary negotiation or an inadequate market size that did not sufficiently motivate bidders. The distinguishing feature of the NVBP lies in the implementation of volume-based procurement, where the volume-price linkage is the central policy metric, setting it apart from previous procurement policies.^5 6^ Under the NVBP, winning bidders are guaranteed a market size of 60%–70% of the total annual drug consumption in the previous year, providing the public payer with significant bargaining power over pharmaceutical manufacturers. The guaranteed quantity also creates economies of scale and eliminates possible supply and demand mismatch, allowing pharmaceutical manufacturers to lower costs.^7^ The large market size also intensifies competition among different pharmaceutical companies. As a result, when a smaller pharmaceutical company wins the tender, it can experience a substantial increase in the size of the market it supplies, providing it with a potential opportunity to transition from a smaller player to a prominent industry participant.

China’s NVBP policy stands out not only for its utilisation of public tendering and pooled procurement strategies but also for its unique approach, which involves the integration of these two methods. Unlike traditional approaches, pooled procurement in China is determined by the volume consumed in the previous year. Furthermore, the procurement process is not undertaken all at once, allowing participating public hospitals the flexibility to make purchases within a specified timeframe rather than at a single point in time. This arrangement ensures that hospitals have the necessary drugs available before the time when the contracted amount should be delivered. Finally, the NVBP occurs on a regular basis, typically two times a year, providing a systematic and scheduled approach to drug procurement. This comprehensive approach also enables the government to closely supervise the actual volume purchased by public hospitals and establishes clear market expectations for pharmaceutical companies. Additionally, the programme is open to all public hospitals in China, which means that even small hospitals in remote areas have access to the same lower drug prices as larger hospitals in urban areas.

### Collaborative reform involving health insurance, healthcare and medications

**Figure 3:**
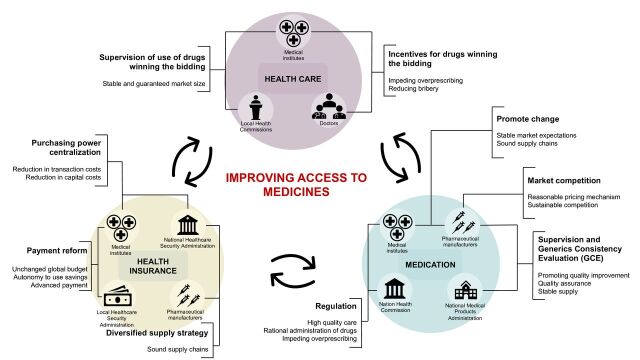
Collaborative reform strategies in the provision of health insurance, healthcare and medications. Source: Self-made by the authors of this study.

The government uses multiple social and policy tools involving the provision of health insurance, healthcare and medications, aiming to establish sustainable competition and collaboration among government, pharmaceutical manufacturers and society. Figure 3 describes the detailed strategies that go into improving access to medicines.

#### Health insurance

##### Purchasing power centralisation

By centralising purchasing power and combining procurement with tendering, the NVBP establishes uniform standards for tendering across different regions, eliminates secondary negotiations and reduces the involvement of intermediaries between manufacturers and public buyers. This centralisation leads to cost savings for pharmaceutical manufacturers, who no longer incur transaction and capital costs associated with the fragmented procurement system.

##### Payment reform

In order to encourage hospitals to use low-priced drugs under the NVBP, the local healthcare security administration has the authority to allocate and maintain the global budget of public medical facilities that is generated from health insurance. This ensures that hospitals receive the same level of funding even if they choose to use the cost-effective bid-winning drugs, thereby mitigating potential resistance stemming from any decrease in income associated with using low-priced drugs. The savings that the hospitals realised from using NVBP drugs should primarily be allocated towards rebalancing the salaries of hospital staff. Directing the savings from using the low-price drugs to doctors can disincentivise overprescribing since the savings will replace the income that they will lose from not prescribing high-priced drugs.

In addition, as part of the implementation of the NVBP, the National Healthcare Security Administration adjusted reimbursement standards for patients upwards to encourage their selection of drugs made by bid-winning manufacturers.^8^ The reimbursement arrangement is based on the price of the NVBP bid-winning drug. Patients who opt for medications produced by bid-winning manufacturers are only required to pay a small portion of the cost, with the remainder covered by health insurance. Reimbursement is also available for drugs produced by non-bid-winning manufacturers. However, patients who choose non-bid-winning drugs are responsible for additional payments to account for the difference between the actual drug price and the NVBP bid-winning drug price. This approach aimed to create a financial incentive for patients to choose the low-cost option available, which aligns with the cost-saving objectives of the NVBP.

In general, about 30% of the total drug procurement cost will be directly transferred from local healthcare security administrations to the medical facilities as an advanced payment, aiming to reduce their financial burden.

##### Diversified supply strategy

A diversified supply strategy is a fundamental feature of the NVBP. To foster sustainable supply-side competition, the NVBP awards multiple winning contracts instead of ‘winner takes all’ contracts, which increases the robustness of supply chains (except for the 4+7 pilot scheme, which implemented a single winner strategy).

#### Health care

##### Usage supervision

All public medical facilities involved in the NVBP are required to give priority to prescribing bid-winning drugs over other more expensive brand name versions or non-bid-winning generic drugs.^9^ The National Health Commission (NHC) has implemented a standardised performance evaluation mechanism for tertiary care public hospitals, which play a crucial role in China’s three-tier healthcare system. In this system, tertiary care hospitals are positioned at the top tier and are designed to provide advanced and specialised healthcare services. The evaluation mechanism developed by the NHC encompasses various aspects of the overall performance of these hospitals, including the utilisation of NVBP drugs, to promote cost-effective medication practices.^10 11^ Hospitals that demonstrate a higher usage rate of NVBP drugs and achieve better scores in their performance evaluation are eligible for financial support and development assistance. The success of these tertiary care hospitals also establishes a gold standard, influencing low-level hospitals to learn from their practices and improve their own performance. The NHC has expressed its intention to expand this evaluation mechanism in the future to encompass a broader range of public hospitals.^10^

##### Incentives

Usually, public medical facilities will provide financial incentives for doctors, patients and hospitals to prescribe and use bid-winning drugs. Incentives for doctors and patients were described in the section about payment reform. Hospitals that successfully meet the target for using bid-winning drugs typically receive higher performance evaluations, resulting in higher budgets from the government. A failure to meet the target can result in financial punishment.

#### Medications

##### 30-day payment term

Public medical facilities are required to pay the cost of purchased drugs within 30 days after the total volume has been supplied. This requirement reduces the financial burden on pharmaceutical manufacturers.

##### Quality supervision

The National Medical Products Administration evaluates the quality of drugs participating in the bidding and provides assurance of their quality for public buyers. See figure 3 for the detailed strategies that go into improving access to medicines.

### Effects of the NVBP

The initial results from the seven rounds of the NVBP have demonstrated its effectiveness in achieving several key objectives since its inception in 2018. It has successfully eliminated kickbacks, reduced corruption in pharmaceutical procurement, facilitated fair competition among suppliers and established a primary market-oriented drug pricing mechanism.^12 13^ As of the end of 2021, the NVBP has saved over 260 billion CNY (approximately US$36.3 billion),^14^ improving the efficiency of health insurance funds that cover part of healthcare costs. From the perspective of patients, the NVBP has improved access to affordable drugs, thereby reducing their financial burden. Additionally, the proportion of quality-assured drugs (generic drugs certified by GCE and originator drugs) used has increased from 50% to over 90% of the total.

In conclusion, the NVBP represents a crucial policy lever for achieving universal healthcare coverage through strategic purchasing of pharmaceuticals.^15 16^

## What is next?

**Figure 4:**
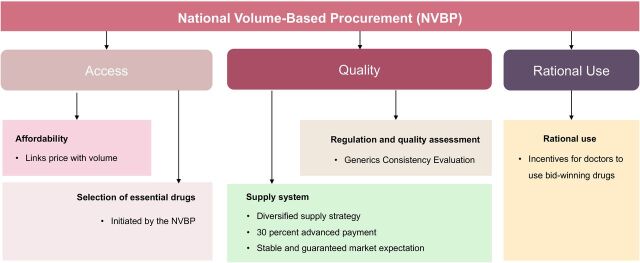
Components of China’s NVBP policy. Source: Self-made by the authors of this study.

China’s experience and efforts highlight the importance of political commitment and collaboration among government, pharmaceutical manufacturers and the public. Unlike typical industrial production, where the end result is a concrete commodity, the end result of the NVBP should not be evaluated in terms of prices or the quantity ordered but should be based on improving health outcomes and ensuring access to essential medicines. Under the framework of the WHO’s National Drug Policy, the NVBP directly improved all three medicine objectives of access, quality and rational use using six key components (see figure 4). We believe that some of these measures, although rooted in Chinese culture, can be transferred to other LMICs. Pooled procurement should be triggered by market size and the importance of specific drugs, particularly essential drugs. A diversified supply strategy is a fundamental requirement to facilitate sustainable competition among pharmaceutical manufacturers, stabilising the market and the supply chain. As LMICs cannot afford to evaluate the quality of all drugs, consistent evaluation of the quality of generic versions of important drugs could be an economical and viable measure to improve the quality of selected drugs. Although the NVBP is workable in a fragmented pharmaceutical system, a robust and strong public drug plan is essential for its generalisability to other LMICs, generating massive purchasing power and compelling suppliers to reduce prices. Furthermore, a powerful government is necessary to centralise purchasing power and create robust market competition.

Since its establishment, the Chinese government has been continuously improving the NVBP in a variety of ways (box 2). However, the NVBP still faces several challenges. The first challenge arises from the boundary between central and local governments. The NVBP centralised the purchasing power across regions of China and reallocated the authority between central and local governments. On the one hand, this restructuring can break down regional markets and reduce regional protectionism in the pharmaceutical market. On the other hand, it deprives local governments of their autonomy in selecting drugs and could lead to resistance and conflict from local governments. In practice, a top-down NVBP potentially provokes resistance to using bid-winning drugs at a local level, among physicians and patients. Physicians and patients may prefer originator drugs that were previously perceived to be more effective or of higher quality than corresponding generic drugs. Even though incentives have been introduced for doctors, health facilities and patients, it is necessary to explore more systematic approaches to boosting public confidence in bid-winning generic drugs and enhance physicians’ and patients’ acceptability of them. Therefore, some argue that the NVBP should become a regular and normalised programme to achieve sustainable price reductions for drugs. On the other hand, some suggest that the NVBP has fulfilled its historical mission and that the central government should end its centralised purchasing power and return purchasing power to local governments. It is difficult to determine which view is correct at this moment, but a pricing mechanism that provides long-term stability is needed in China. This mechanism could be the NVBP or involve designing another policy.

Box 2 **Improvement in national volume-based procurement (NVBP) policies**

**Expansion of NVBP**

- The NVBP was initially piloted in 11 cities, then expanded to 25 provinces, before being rolled out across the country.
- The NVBP began with chemical drugs that were relatively suitable for generics consistency evaluation (GCE) and gradually expanded to include biosimilars, proprietary Chinese medicines and medical devices.

**Bid-winning rule refinement**

In the first round of the NVBP, the pharmaceutical company with the lowest price would be the sole winner. In order to implement a diversified supply strategy, in subsequent rounds of the NVBP, the bid-winning rule has continuously improved:

- Any supplier who offers a price that is no more than 80% above the lowest-offered price among all participant pharmaceutical companies is also eligible to supply a portion of the market (50% for the sixth round of the NVBP, which is specific for insulin drugs).
- There is no less than a 50% price reduction compared with the maximum allowable price (40% for the sixth round of the NVBP, which is specific for insulin drugs).
- Unit comparable price of any supplier that is no more than 0.1 RMB above the lowest-offered price among all participant pharmaceutical companies is also eligible to supply a portion of the market.
- Instead of a single winner, there are multiple winners (up to 10).
- NVBP will be triggered in the situation where there are no less than four manufacturers who have obtained GCE certificates for a particular drug.

Another challenge for the NVBP is related to its policy objectives. The main objectives of the programme include reducing drug prices and patients’ financial burden, reducing transaction costs, improving the pharmaceutical industry’s functioning, improving drug use, supporting the reform of public hospitals and exploring a bulk purchasing mechanism. However, too much focus has been placed on price reduction, and many people now take a 50% reduction in drug prices for granted. Obviously, drug prices cannot continuously decrease and must be sustainable for pharmaceutical manufacturers. Prices that are too low might disincentivise pharmaceutical manufacturers and reduce appropriate investments that are necessary to ensure a reliable supply and to develop new drugs. The question is whether the NVBP is still necessary if it achieves no price reductions in the future. It is essential to strike a balance between price reduction and the sustainability of the pharmaceutical industry, as both are crucial for the long-term success of China’s drug policy.

The present article mainly focuses on the initial rounds of the NVBP. Further research is necessary to examine the long-term effects of the NVBP on the pharmaceutical industry, particularly with regard to whether it has promoted the development of therapeutically innovative new drugs that enhance healthcare and improve public health by promoting innovation, improving management and lead to the development of higher quality drugs. In addition, by promoting the use of generic drugs, the patent cliff has become more of a concern for drug manufacturers incentivising them to become more efficient. It is also essential to assess whether the reduction in drug prices can be maintained over an extended period. From affordability to quality, China still has a long way to go in refining its drug policy.

## Data Availability

All data relevant to the study are included in the article.
